# Evaluation of the effectiveness of a joint general practitioner-pharmacist intervention on the implementation of benzodiazepine deprescribing in older adults (BESTOPH-MG trial): protocol for a cluster-randomized controlled trial

**DOI:** 10.3389/fmed.2023.1228883

**Published:** 2023-08-25

**Authors:** Jean-François Huon, Pierre Nizet, Pascal Caillet, Hélène Lecompte, Caroline Victorri-Vigneau, Jean-Pascal Fournier, the BESTOPH-MG consortium, Laurent Flet, Erwan Corbineau, Alexandra Gallin-Castagne, Maxime Lebeaupin, Leïla Moret, Émilie Guegan, Pascal Artarit, Valéry-Pierre Riche

**Affiliations:** ^1^Nantes Université, Univ Tours, CHU Nantes, CHU Tours, INSERM, MethodS in Patients-centered outcomes and HEalth Research, SPHERE, Nantes, France; ^2^Nantes Université, CHU Nantes, Pharmacie, Nantes, France; ^3^Public Health Department, Nantes Université, CHU Nantes, Nantes, France; ^4^Centre d'évaluation et d'information sur la Pharmacodépendance-Addictovigilance, CHU Nantes, Nantes, France; ^5^Département de Médecine Générale, Université de Nantes, Nantes, France

**Keywords:** primary care, collaborative practice, implementation, deprescribing, benzodiazepines

## Abstract

**Background:**

Deprescribing benzodiazepines and related drugs (BZDR) is a challenge due to a lack of time on physicians’ part, a lack of involvement of other health professionals, and the need for adapted tools. This study is based on primary care collaboration, by evaluating the effectiveness of a joint intervention between general practitioners and community pharmacists on the implementation of BZDR deprescribing in older adults.

**Methods:**

This is a cluster randomized controlled trial in which each cluster will be formed by a physician-pharmacist pair. Within a cluster allocated to the intervention, the pharmacist will be trained in motivational interviewing (MI), and will offer the patient 3 interviews after inclusion by the physician. They will base their intervention on validated deprescribing guidelines. The pharmacist will receive methodological support during the first interviews. Interprofessional collaboration will be encouraged by writing reports for the physician after each interview. The following implementation outcomes will be evaluated: acceptability/adoption, appropriateness, cost, and fidelity. They will be measured by means of sociological interviews, observations, logbooks, and cost-utility analysis. Focus groups with physicians and pharmacists will be carried out to identify levers and barriers experienced in this collaboration. Observations will be conducted with pharmacists to assess their approach of the MIs. Effectiveness outcomes will be based on medication (discontinuation or reduction of BZDR) and clinical outcomes (such as quality of life, insomnia or anxiety), assessed by health insurance databases and validated questionnaires.

**Discussion:**

This study will determine whether collaboration in primary care between physicians and pharmacists, as well as training and coaching of pharmacists in motivational interviewing, allows the implementation of BZDR deprescribing in the older adults.

This study will provide an understanding of the processes used to implement deprescribing guidelines, and the contribution of collaborative practice in implementing BZDR discontinuation. The cluster methodology will allow to assess the experience of the relationship between the different primary care actors, and the related obstacles and levers.

The results obtained will make it possible to produce guidelines on the involvement of community pharmacists in the management of substance abuse in older adults, or even to legislate new missions or care pathways.

**Clinical trial registration:**

ClinicalTrials.gov, identifier, NCT05765656.

## Background

According to a 2017 report from the French National Agency for the Safety of Medicines and Health Products (ANSM), 13.4% of the French population used a benzodiazepine or related drug (BZDR) at least once in 2015 (1). These drugs are consumed for hypnotic or anxiolytic purposes in most cases. As per the recommendations, BZDR should not be prescribed for more than 28 days when for hypnotic use and for 8 to 12 weeks, including withdrawal, when for anxiolytic purpose. Indeed, these drugs have shown a real, but mediocre, short-term efficacy on anxiety and sleep disorders (2). Moreover, their long-term effectiveness is almost nil. However, literature shows that nearly one patient out of six taking a BZDR is a long-term user (3) and that the proportion of patients for whom the indication is questionable can reach 2/3 (4). The consequences of BZDR are multiple with an increased risk of daytime sedation, balance disorders leading to falls and fractures, cognitive disorders, road accidents and dementia (1, 2). Also, given their comorbidities, physiological changes, and multiple medications, the older adults are more at risk of experiencing from BZDR adverse events, like falls, driving accidents, dementia or even death (5–9). The majority of patients are unaware of these potential risks and continue to use these medications over the long term. They overestimate the benefits of BZDR and underestimate their harmful effects. The consequences are substantial, both from a health and financial perspective: in France, BZDR represent more than 4% of total drug consumption (10), and 117 million euros in sales (excluding taxes) per year.

At the national level, numerous actions have been taken by the health authorities to reduce the use of BZDR: information for health professionals, pictograms on drug boxes, recommendations by health authorities, incentive measures, and regulatory measures. However, despite these numerous initiatives, the consumption of BZDR remains high. Their deprescribing, defined as discontinuing or reducing under the supervision of a health care professional the dose of medications that are no longer needed, for which risks outweigh the benefits (11, 12) has difficulties being implemented in real life.

Literature shows that many levers can facilitate the implementation of actions for the appropriate use of drugs. Especially, interprofessional collaboration has shown efficacy in improving prescribing appropriateness and affect patients outcomes positively (13–15). As concluded by Nurchis et al., policy makers should promote the widespread adoption of a collaborative approach (15). This may address the discomfort of general practitioners (GPs) who report not feeling fully capable of implementing interventions to deprescribe BZDR if they have to rely solely on guidelines (16), and because of issues such as lack of time to re-assess these treatments, availability of mental health resources, and multiplicity of prescribers (17, 18). Yet, current international deprescribing studies remain mainly based on actions only directed at the prescriber (19), whereas collaboration between primary care professionals appears to be a solution for implementing a decision to stop treatment (17). In addition, BZDR users are very often described as reluctant to stop their medication for fear of a return of anxiety or insomnia (20). In this context, another lever usable to achieve the implementation of deprescribing is the use of techniques that enhance patients’ motivation to change and their engagement in the intervention proposed by their GPs. As such, motivational interviewing (MI) may reduce the extent of substance abuse compared to no intervention (21). Developing and promoting training for healthcare professionals in MI may be a simple and pragmatic implementation strategy to reduce inappropriate BZDR use.

For this study, an interprofessional collaboration between the GP and the pharmacist will be held within primary care setting, with a specific training of pharmacists in MI, allowing the implementation of BZDR deprescribing in the older adults. The acronym BESTOPH-MG stands for “*BEnzodiazépines STOp PHarmacien Médecin Généraliste”(=* “*BEnzodiazepines STOp PHarmacist General Practitioner”).* Clusters composed of a general practitioner and community pharmacist pair will be made and both cluster and patient level objectives will be addressed. The specific aims are (1) to examine implementation outcomes like patient’s factors of receptivity to the intervention, effects of the program on the professional practices of GPs and pharmacists, or reduction in health care consumption, economic efficiency and effectiveness of the intervention on inappropriate BZDR deprescribing rate, and (2) to evaluate if this intervention is associated with improvement in patients reported outcomes and use of other substances following the cessation of BZDR.

## Methods

### Design and setting

**Figure 1:**
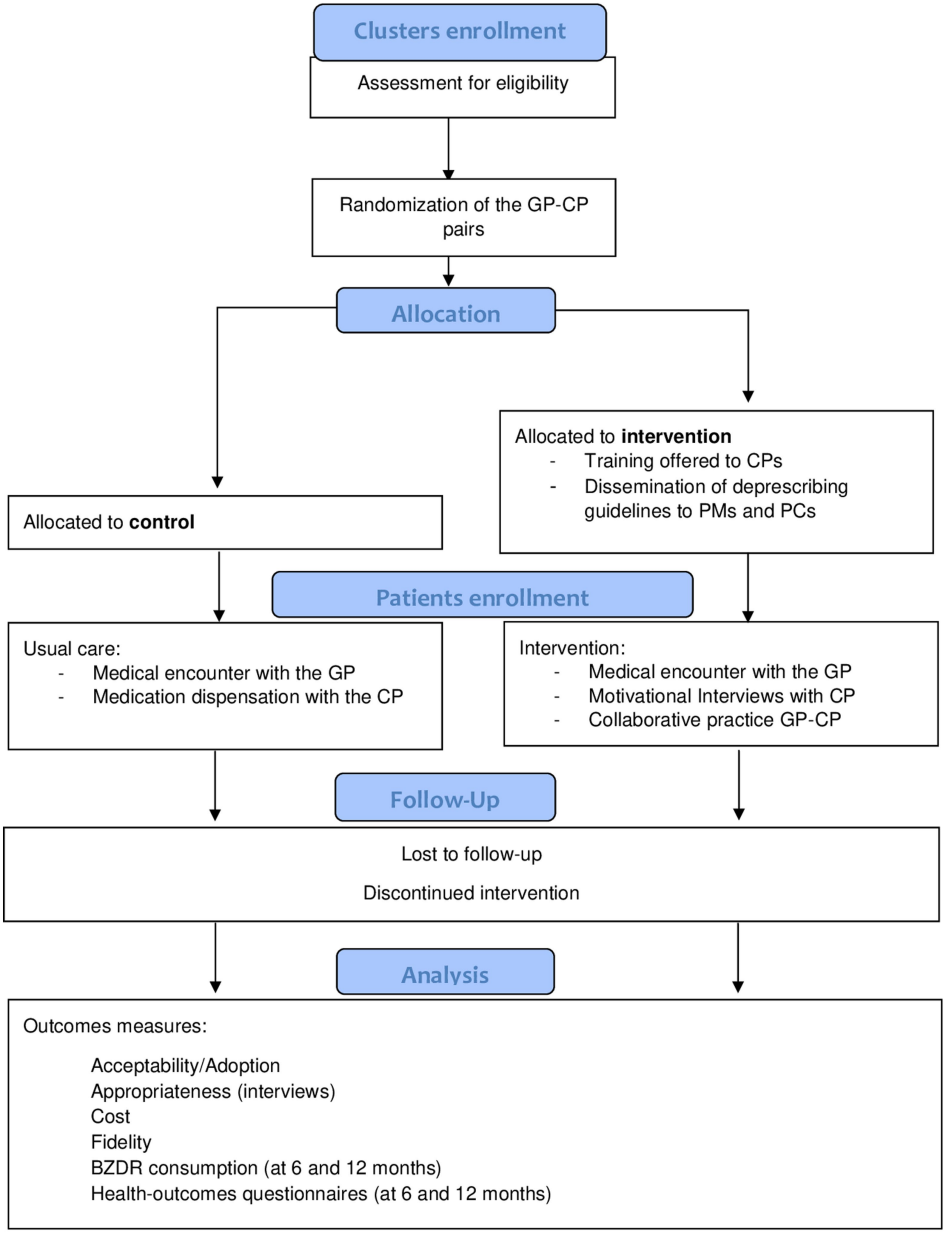
Study flow chart.

This is an open cluster-randomized pragmatic trial with parallel groups and conducted in primary care setting. The study is led by the Nantes University Hospital and is being conducted in the “Pays de la Loire” Region of France. Each cluster will be made up of a GP-Community Pharmacist pair (GP-CP), both of whom already have regular professional exchanges about patients, in order to respect an existing territorial organization. A clustered design was chosen to avoid potential contamination between the intervention and control arms. Indeed, a GP-CP pair applying both the intervention and control condition could lead to patients from the control condition being treated as if they were in the intervention condition. The GP-CP pairs will be randomized with a 1:1 ratio (intervention/control), and all patients handled by the pair will receive the same intervention. If necessary, several physicians from the same practice will be grouped together in the same cluster, and associated with the same pharmacy, in order to be randomized in the same arm and avoid potential contamination between physicians. Figure 1 illustrates the flow chart.

### Study population and recruitment

#### Patients

In order to minimize the risk of subjective pre-selection of patients by the GP, a predefined list of eligible patients per cluster will be sent out to each GP by the Health Insurance according to the inclusion criteria detailed below, before randomization of the clusters and on the basis of the GP’s request. The study population will consist of patients aged 65 years and older, monthly users of at least one anxiolytic or hypnotic BZDR for more than 3 consecutive months (ATC classes N05BA, N05CD and N05CF). Patients meeting the eligibility criteria will be recruited by GPs during their medical encounters.

The inclusion criteria will be: outpatients aged 65 and over, followed by the GP and being delivered their medications by the pharmacist of the GP-CP pair, having a prescription for an anxiolytic or hypnotic BZDR prescribed at least 4 times in the past year, the last prescription being dispensed in less than 3 months and having been dispensed monthly during the last 3 months, affiliated to a social security scheme, and having given consent to participate in the research.

Non-inclusion criteria will be: patients living in an institution, participating in another clinical trial, with any medical condition that contraindicates BZDR discontinuation on the physician’s opinion, unable to participate in an interview or answer a questionnaire and with insufficient autonomy to carry out the steps inherent in the study. Patients living in an institution will be excluded because an unknown part of them have their BZDR not traced in the SNDS database (for administrative reasons), which could induce bias in our study.

If eligible, the patient will be offered to enroll in the study during a standard medical consultation. If he agrees to participate, signed informed consent will then be obtained, and the patient will be assigned a patient number automatically upon inclusion.

#### Primary healthcare professionals

This study will involve GPs and pharmacists practicing in primary care, i.e., in medical practices or community pharmacies. GP and CPs in the Pays de Loire region will be contacted by e-mail through multiple channels like research networks and professional associations. An evening webinar regarding BZDR misuse will be held for physicians and pharmacists in the Region. The objective will be to raise awareness of the problem and to explain the study to professionals. If a GPs shows interest, he/she will be asked to reach out the pharmacist (or vice versa) in order to form a pair (cluster). Pre-existing interactions between healthcare professionals of the same area is a major driver regarding the optimal implementation of the intervention within the cluster. Moreover, as in the context of a pragmatic trial, this way of conducting recruitment is the most coherent regarding the evaluation of what would be observed in practice when deploying the intervention to a larger scale. If both GP and CP agrees to participate, signed informed consent will then be obtained, and the cluster will be randomized.

### Randomization

A schedule for enrollment, randomization and allocation, interventions, and assessments for the study is presented in Table 1. The randomization will be performed by cluster of GP-CP pairs in open without stratification with a ratio of 1:1 using the *Ennov clinical* software via a secure connection to the servers of the Nantes University Hospital. The information necessary for communication will be provided to the recruiting GPs by a data manager of the Nantes University Hospital. The randomization will be computer-generated by blocks of 6 as the clusters are recruited. The trial statistician and the researchers assessing the outcomes will be blinded to the randomization results.

**Table 1:** Schedule of enrollment, interventions, and assessments.

	2022	2023			2024		
	June-Dec.	Jan.-Feb.	March-Sept.	Oct.-Dec.	Jan.-March	April–June	July-Sept.
Enrollment							
Recruitment and GP-CP clusters forming	X	X	X				
Eligible patients’ identification			X				
Clusters allocation		X	X				
Intervention							
CP training in MI performing		X	X				
Patients enrollment and MI 1 to 3			X	X			
Monthly reports to GP			X	X	X		
Assessments							
Acceptability/Adoption		X	X				
Appropriateness (interviews)				X		X	
Cost						X	
Fidelity			X	X	X	X	
BZDR consumption				X	X		
Health-outcomes questionnaires				X	X		
Dissemination							X

### Intervention

Deprescribing will be implemented through 3 successive steps, the cornerstones of which are interprofessional collaboration between GPs and CPs, and training and methodological support for CPs.

#### Step 1: medical encounter with the GP and submission of documents

Patients in the GP-CP clusters randomized to the intervention arm will be offered a joint GP-CP deprescribing intervention by their GP. The physician will present the study schedule to the patients and inform them of the planned follow-ups.

#### Step 2: medication treatment dispensing and MI

Following the medical consultation, the patients will go to the pharmacy to get their medication treatment dispensed. They will be given a kit of patient education materials addressing the risks of BZDs and the benefits of reducing/stopping their use. This kit will be designed on the basis of patient education materials produced by the Canadian Deprescribing Society^1^ and adapted to the French context. The pharmacist will then plan with the patients 3 MIs to be carried out within the next 3 months. These interviews will last 30 to 60 min and will address the risks of using BZDR, as well as the benefits and modalities of stopping them. MI is a client-centered, semi-directive method for enhancing intrinsic motivation to change by exploring and resolving ambivalence (22). It is intended to work through four main principles: express empathy, support self-efficacy, roll with resistance, and develop discrepancy. During these interviews, the pharmacist will provide information about non-pharmacological ways to improve sleep or reduce anxiety, prevention of rebound phenomena, and will provide an example of de-escalation of doses (i.e., progressive reduction). The pharmacist will identify the patients’ representations of their medication and pathology (insomnia, anxiety), and will understand the barriers and facilitators for deprescribing. Any question the patients may have will be addressed. The choice to stop or reduce the dose of the BZDR will be the result of a shared medical decision (trio patient – GP – PC). To facilitate implementation, the pharmacists of the intervention arm will receive a 2-day specifically designed training course in MI by a certified organization prior to the intervention (Table 2). They will be given guidelines on the risks associated with BZDR, their deprescribing, and the management of patients who were deprescribed. If required, the pharmacists will be supported by the interventional public health department of the promoting center which will accompany them in their first MI. To avoid contamination, pharmacists working in pharmacies randomized in the control group will not be trained in MI and in deprescribing until after the study is completed.

**Table 2:** Objectives and program of the pharmacist’s training course in Motivational Interview.

Pedagogical objectives	
To discover Motivational Interviewing (MI) To become imbued with the spirit of the MI and to identify the corrective/repairing reflex To Mobilize the skills of the MI To use specific tools to share information in a motivational way To identify the change discourse To begin to focus questions and reflections to promote discourse-change To practice and receive feedback on MI practice in a training context	
Pedagogical means	
The training uses a variety of media (video, summary document, presentation, written exercises, workshops and oral role plays, brainstorming, exchanges between participants)	
Training program	
Day 0	Reading of a summary of MI: history, key elements of MI and definitions
Day 1	Creating the group dynamic – introduction to Engagement in MI Review of theory Ambivalence The corrective/repairing reflex Introduction to empathy and reflective listening Open questions Summarizing DDPD tool (motivational information sharing) Valuation Practice around a polycephalous interview (with several people) to building the alliance and engaging the relationship Conclusion
Day 2	Renewal of group dynamics and commitment The different types of simple and complex reflections Introduction to sustaining and changing discourses The different types of discourse-change Introduction to evocation strategies Observation of a live MI by the trainer on the subject of deprescribing BZDR Practice of a MI by each participant and feedback from the trainer with the elements congruent with the MI and one or two possible areas for improvement Conclusion and evaluation

#### Step 3: interprofessional communication

Following each interview, the pharmacist will inform the physician by means of a formalized report of the points discussed and of the relevant information concerning the objective of deprescribing (barriers, facilitators, etc.), using the patient’s comments when appropriate. The report will include the data collected from the patient during the interviews (expressed needs) as well as the pharmacist’s conduct (initiation of de-escalation, associated advice, dispensing of therapeutic alternatives). The pharmacist will inform the GP of the patient’s choice or not to get involved in a deprescribing process and of the protocol followed, if applicable. The objective of this exchange is to formalize the joint GP-CP intervention and to secure the deprescribing of BZDR for the patients.

Patients included in the control group clusters (non-interventional) will not benefit from the joint GP-CP intervention but will be managed in the usual way: medical consultation followed by dispensing of medication treatments. Blinding of participants for this type of intervention is not possible.

### Data sources and collection

The implementation outcomes will be measured by different procedures detailed below (see Outcomes section).

The sociological study will consider the variability of practice locations to be more representative: rural, urban, peri-urban. Depending on the location of the practice, the patient may have different social characteristics that will probably require professionals to adapt (vocabulary, arguments, conduct of the interview, etc.). Four days of observations will be conducted with pharmacists who have just been trained in MI to study, in action, how they conduct their first interviews with the older adults. These same pharmacists will be observed a second time at the end of the study, to see how their approach to MI has evolved.

A first wave of ten semi-structured interviews will be conducted with older adults who have already been seen by their pharmacist, to see what effects the pharmacist has had on their representations of BZDR and on their consumption.

Finally, three focus groups will be carried out, one with CPs, one with GPs and one with pairs. The first two will allow to identify any difficulties experienced in this interprofessional collaboration, but also what has been facilitated. The last focus group will allow the pairs to exchange and compare their point of view on multidisciplinary work and relevance of the collaboration.

Regarding the effectiveness of the intervention, the consumption of reimbursed drugs and the co-payments of the system will be assessed using the National Health Data System (SNDS) (23). This database provides access to all reimbursed healthcare, including drugs dispensed in pharmacies.

The outcomes concerning the clinical evolution of the patients will be measured using questionnaires administered to the patients by telephone by a clinical research associate.

## Data analysis

### Outcomes

#### Aim 1: implementation and effectiveness outcomes

Implementation outcomes will be assessed according to Proctor et al. classification (24) as described below: acceptability/adoption, appropriateness, cost and fidelity (Table 3). All qualitative data will be transcribed in full before being analyzed. The interviews and focus groups will be subject to thematic and structural discourse analysis. No software will be used.

**Table 3:** Implementation and effectiveness outcomes.

	General Practitioners and Community Pharmacists	Patients
Implementation outcomes		
Acceptability/Adoption	Nb of clusters included/Nb of clusters planned Reason for refusal	Nb of patients included/Nb of patients eligible Factors of receptivity
Appropriateness	GP & CP Sociological interviews CP Observations	Sociological interviews
Cost	Cost-Utility and Budgetary impact analysis	
Fidelity	Nb of clusters completing the study/Nb of clusters Rate of CP’s reporting to GP	Nb of appointments/Nb of planned appointments
	Nb, duration and frequency of MI	
Effectiveness outcome		
Medication		Cessation or reduction of BZDR at M12
Clinical		Patient reported outcomes

CP, Community Pharmacists; GP, General Practitioners; MI, Motivational interview; Nb, Number.

##### Acceptability/adoption

The achievement of the number of clusters and patients included planned in the protocol will be evaluated. A short questionnaire will be sent to a sample of GPs and pharmacists who refused to participate in order to understand the reasons for not participating in the study.

The patients’ factors of receptivity to the intervention (gender, age, couple, socio-economic level, last diploma, literacy level, frequency of consultations with the GP, duration of prescription, indications for BZDR) will be studied in order to identify a typical profile of the patient responding to the intervention.

##### Appropriateness

The sociological study will allow to analyze the appropriateness of this work in pairs on the deprescribing of BZDR among the older adults. We will study the impact of this collaboration on GPs and CPs, and in particular analyze the extent to which it allows for an increase in the competence of the professionals. Based on a field survey that will put the discourse (interviews) into perspective with the practices (observations), the effects of this arrangement on all parties and the effectiveness of this collaboration will be assessed, while identifying how each party has lived this experience, what they have learned and any resistance to change. In this way, how pharmacists use motivational interviews, how they conduct them, and what this multidisciplinary collaboration brings them will be studied, but also how they transmit new norms to the older adults and the concrete effects of these motivational interviews.

Furthermore, by observing the way in which these motivational interviews are constructed and conducted according to the different practitioners, and by putting these practices into perspective with the discourses of the doctors and pharmacists, we will be able to question the appropriateness of this multidisciplinary vision, and how it upsets the socialization and professional logics of the two.

##### Cost-utility

Economic efficiency of the implementation effort will be assessed following the HAS 2020 recommendations (25). A Cost-Utility Analysis (CUA) expressed as a cost per Quality Adjusted Life Year (QALY) will be performed from a collective perspective and with a time horizon of 12 months. The CUA will consider the direct costs of care from randomization to M12. Hospital and city care consumption data related to the management and its consequences will be collected in both arms via the Health Insurance database (SNDS). The data collected will include: medications, medical consultations, hospitalizations if applicable, and emergency room visits. A budgetary impact analysis from the point of view of the Health Insurance will be carried out following the CUA. It will be based on different scenarios of 5-year diffusion levels of the joint intervention.

##### Fidelity

The proportion of pairs completing the study, the proportion of patients who actually made appointments with the pharmacist, the number, duration and frequency of MI, and the rate of reporting made by the pharmacist to the GP will be measured through a logbook.

##### Effectiveness

The effectiveness will be assessed by the cessation or reduction of BZDR use at 12 months from inclusion. It will be described as the proportion of patients no longer being dispensed BZDR at 10 months after inclusion, with the last two months (10 to 12 months) not counted to account for possible residual BZDR use, or having decreased their average Daily Drug Dose (DDD) dispensing by 50%. The decrease will be calculated by comparing the average DDD dispense over the last 3 months of patient follow-up to the dispense observed during the 3 months prior to inclusion.

#### Aim 2: patient reported outcomes

Secondary outcomes will aim to evaluate improvement in patients reported outcomes, and use of other substances. Patients’ quality of life (measured by EQ5D-5L), anxiety disorders (GAD-7), quality of sleep (ISI), time to first hospitalization after inclusion (SNDS), occurrence of falls, dependence on BZDR (ECAB), autonomy (IADL) and use of other substances will be assessed.

A stratified analysis by type of BZDR (hypnotic or anxiolytic) will be carried out on each of the outcomes to determine the impact of the indication on the implementation elements.

### Sample size and power

The calculation of the number of subjects needed is based on the effectiveness outcome, on the basis of the following assumptions: risk α equal to 5%, statistical power equal to 80, 5% deprescribing proportion in control group based on current trend, a proportion of at least 15% in the intervention group, an intraclass correlation coefficient of 0.05 (26) and a number of 20 GP-CP pairs per arm. The number of patients to be recruited will be 400 (200 per arm), based on the inclusion of 10 patients per GP-CP pair, with 20 GC-CP pairs per arm during the 12 months of inclusion (27). To reach target sample size, various networks will be involved in recruitment: primary care research network, professional associations, regional unions of practitioners, university training masters.

### Statistical analysis

Variables measured at inclusion will be described according to the randomization group for all included patients. Quantitative variables will be described using the mean, standard deviation, quartiles and range. Qualitative variables will be described using the numbers and proportions for each modality.

In order to measure the impact of the intervention in real life, the analysis will focus on the intention-to-treat population: all patients included will be kept in the analysis sample according to their randomization group, regardless of protocol deviations.

For the effectiveness endpoint, a mixed logistic model will be built to study the proportion of deprescribing between MI condition (intervention) and usual care condition (control), and concomitantly accounting for the cluster effect associated with our design (28). For secondary analysis, the impact of the intervention on quality of life, anxiety disorders, sleep quality, dependence and autonomy will be estimated by comparing the EQ-5D-5L, GAD-7, ISI, ECAB, and IADL scores between the two groups using mixed models. As advocated by the literature (29), models will be adjusted on baseline covariates that are prognostic of the outcome, balanced or not at baseline, including *a minima* the following: age, sex, number of past attempts, suspected addiction. The impact of the intervention on hospitalizations and deprescribing failures will be estimated from hospitalizations (number of hospitalizations) and failures (number of discontinuations and then resumption).

## Discussion

The objective of this trial is to assess the added value of a primary care collaboration between GPs and CPs in order to implement inappropriate BZDR deprescribing in older adults. In various countries, legislative and regulatory developments have led to an expansion of the scope of practice of pharmacists for the substitution or discontinuation of certain medications, including BZDR (30). The patient-centered intervention developed in this study aims to reinforce known levers and overcome barriers to stopping inappropriate medications.

Wei et al., in a 2022 meta-review, reported that improving interprofessional collaboration requires organizational, teams, and individuals’ combined efforts but that when effective collaborations occur, all stakeholders can benefit – organizations, professionals, and patients. Our study is grounded in a concrete way on existing collaborations. It strengthens the bond between physicians and pharmacists who work together on a daily basis. This pragmatism is a major implementation tool when setting up studies in primary care.

The use of MI techniques have demonstrated their effectiveness in various studies (31, 32) and allows us to hypothesize that this intervention will be beneficial to a patient who is initially reluctant to stop or reduce his or her consumption of BZDR. Indeed, the literature suggests that MI is a powerful tool for BZDR deprescribing, as it is popular with healthcare professionals (33) and has been shown to be effective with other drugs such as opioids (34). A recently published review (35) described that different interventions had a positive impact as soon as they were based on patient empowerment (36–38). MI, for this purpose, is based on taking into account the ambivalence a patient may have about taking action. This ambivalence is well described: patients are aware of the benefits of stopping their BZDR (perception of long-term side effects, burden of treatment, desire for natural sleep), but face inner barriers that block initiation or persistence of cessation (fear of withdrawal effects or return of pathology) (39). Perceived self-efficacy, which may be due to a previous failure to quit, or a lack of knowledge about how to cut down, may also come into play (40). All of these factors provide fertile ground for the implementation of MI in the deprescription of BZDRs in our study. The expected results are a decrease in BZDR consumption for patients in the intervention arm compared to the control group. In addition, this study could show an improvement in cognitive functions (41–43), an improvement in the quality of life of patients (32, 44, 45) and an economic benefit of the deprescribing intervention. Training pharmacists in MI through a specific two-day hands-on training should improve the adoption of the intervention by health professionals. Moreover, as this technique can also be used on other occasions (smoking cessation, therapeutic adherence, etc.), we believe that it is a lever for the involvement of pharmacists in the trial and for the development of a more effective approach. In addition, accompanying CPs during the first interviews should be important levers for the implementation of the MI.

Patient empowerment is a key mechanism for increasing responsibility for shared decision-making with health care providers (46). It is an effective strategy when it comes to deprescribing (47) combined with behavior change strategies and progressive de-escalation. Patient empowerment, in which patients strengthen their ability to effectively care for themselves, has been shown to be a powerful transformative process (31), and giving them educational material may promote deprescribing conversations (48). Combined with direct education, these methods have been shown to be effective in reducing inappropriate use of BZDR in the older adults (49). This tripartite approach for pharmacists, physicians and patients aims to achieve a synergistic impact.

### Strengths

Strengths of the study include its pragmatic design that will allow the observed process to reflect real-world practice as accurately as possible. This pragmatic approach involves intervention at the group level, rather than the individual level, and as such cluster randomizations are the most common (50). Furthermore, this cluster-randomized design is considered the most appropriate for conducting deprescribing trials (51). To our knowledge, this is the first deprescribing trial in France involving interprofessional collaboration of GPs and pharmacists in primary care. Multiple implementation outcomes will allow us to assess how this intervention can be scaled up beyond the clinical trial. Indeed, the qualitative study will allow us to explore the appropriateness of the program by assessing the experiences and representations of the pairs and patients concerning deprescribing. This part will highlight the obstacles and levers to intervention. Finally, an objective assessment of BZDR discontinuation rates from the National Health Insurance database will increase the internal validity of the study.

### Limits

Despite these strengths, our trial has several limitations. Firstly, neither the patients nor the GP-CP pairs will be blinded due to the pragmatic design of the trial (52). However, the statisticians and the clinical research officer will be blinded to reduce the risk of confirmation, monitoring and evaluation bias. Secondly, it is likely that the pairs agreeing to participate in the study will be health professionals already convinced by the deprescribing approach, which is why this study is randomized and controlled. In addition, although pharmacists will be trained in MI by a certified organization, some may be more or less comfortable than others and the implementation of the intervention may depend on the operator of the training. This will be measured by the fidelity of the study to the protocol. Finally, a limitation of this study is that the evaluation of a multifaceted intervention prevents the determination of the specific component(s) responsible for the observed outcomes or failures at the conclusion of the study.

## Conclusion

This study will provide evidence on the effectiveness of a joint GP-CP intervention to aid benzodiazepine deprescribing. If so, this strategy and intervention could be implemented more widely and also for other potentially inappropriate drugs in older people.

## Ethics statement

Ethics committee (CPP Nord Ouest 1) reviewed and approved the study protocol on December 1st, 2022 (22.03226.000131).

## Author contributions

J-FH and J-PF designed and piloted the study. PN co-piloted the study and wrote the first draft of the manuscript. HL is the team’s expert in qualitative evaluation. CV-V contributed to the methodology, in particular the outcomes issues. PC contributed to the selection of methods, analysis plan, and preparation of manuscript. All authors have read and approved the submitted version and have agreed to be personally accountable for their own contribution.

## The BESTOPH-MG consortium

Laurent Flet, Nantes Université, CHU Nantes, Pharmacie, Nantes, France. Erwan Corbineau, Nantes Université, CHU Nantes, Pharmacie, Nantes, France. Alexandra Gallin-Castagne, Nantes Université, CHU Nantes, Pharmacie, Nantes, France. Maxime Lebeaupin, Public Health Department, Nantes Université, CHU Nantes, Nantes, France. Leïla Moret, Public Health Department, Nantes Université, CHU Nantes, Nantes, France. Émilie Guegan, Département de Médecine Générale, Université de Nantes, France. Pascal Artarit, Medical Department, French National Health Insurance, DRSM, Nantes, France. Valéry-Pierre Riche, Service Evaluation Economique et Développement des Produits de Santé, Département Partenariats et Innovation, Centre Hospitalier Universitaire de Nantes, Nantes Université, Nantes, France.

## Funding

This work was funded by a French Ministry of Health grant (PREPS, *Programme de recherche sur la performance du système des soins*). Grant/Award No. PREPS-20-0327 (567 711€). The funding body did not have any role in the design of the study, the collection, analysis, and interpretation of data or in writing the manuscript.

## Conflict of interest

The authors declare that the research was conducted in the absence of any commercial or financial relationships that could be construed as a potential conflict of interest.

## Publisher’s note

All claims expressed in this article are solely those of the authors and do not necessarily represent those of their affiliated organizations, or those of the publisher, the editors and the reviewers. Any product that may be evaluated in this article, or claim that may be made by its manufacturer, is not guaranteed or endorsed by the publisher.

## Footnotes

^1^Deprescribing.org

## Abbreviations

BZDR: Benzodiazepines and related drugs
CUA: Cost-utility analysis
CP: Community pharmacist
DDD: Daily drug dose
GP: General practitioner
HAS: Health high authority
MI: Motivational interview
QALY: Quality adjusted life year
SNDS: National health data system
